# TMEM119 facilitates ovarian cancer cell proliferation, invasion, and migration via the PDGFRB/PI3K/AKT signaling pathway

**DOI:** 10.1186/s12967-021-02781-x

**Published:** 2021-03-17

**Authors:** Tianshui Sun, Fangfang Bi, Zhuonan Liu, Qing Yang

**Affiliations:** 1 Department of Obstetrics and Gynecology, Shengjing Hospital of China Medical University, No. 36, Sanhao Street, Heping District, Shenyang, 110004 China; 2 Department of Urology, First Hospital of China Medical University, Shenyang, China

**Keywords:** Ovarian cancer, TMEM119, Proliferation, Invasion, Migration

## Abstract

**Background:**

Ovarian cancer (OV) is the deadliest gynecological cancer. Transmembrane protein 119 (TMEM119) has been reported as oncogene in several human cancers. However, the function of TMEM119 in OV is still poorly known.

**Methods:**

Western blot and qRT-PCR were used to analyze TMEM119 levels. Transwell assays, wound healing assays, CCK-8 assays and EdU cell proliferation assays were designed to explore the function and potential mechanism of TMEM119 in malignant biological behaviors in OV.

**Results:**

TMEM119 was observed to be overexpressed in OV tissues and associated with poor survival in OV patients. Knockdown and overexpression experiments demonstrated that TMEM119 promoted proliferation, invasion, and migration in OV cells in vitro. TMEM119 mRNA expression was related to the pathways of focal adhesion according to Gene Set Enrichment Analyses and was correlated with the mRNA expression level of platelet-derived growth factor receptor beta (PDGFRB). TMEM119 exerted oncogenic effects partially by regulating the expression of PDGFRB and by activating the PI3K/AKT signaling pathway.

**Conclusions:**

Collectively, our findings highlight the potential role of TMEM119 in the malignant biological behavior of OV, which may serve as a potential biomarker and a therapeutic candidate for OV.

**Supplementary Information:**

The online version contains supplementary material available at 10.1186/s12967-021-02781-x.

## Background

As the deadliest gynecological cancer, ovarian cancer caused 295,414 new cases and 184,799 cancer-associated deaths globally in 2018 [1, 2]. About 70% of ovarian cancer patients are diagnosed at an advanced stage because of lacking typical symptoms and reliable early detection methods [3]. The five-year overall survival rate of ovarian cancer patients is less than 45%, without significant improvement in the past two decades [4]. Clarification of key mechanisms underlying the occurrence and development of ovarian cancer is urgently needed to identify effective tumor biomarkers for early diagnosis and to provide new therapies to improve prognosis.

Existing in biological membrane, transmembrane proteins (TMEMs) are found to be associated with various cancers, including ovarian cancer [5–8]. TMEM158, TMEM45A and TMEM49 have been observed to play oncogenic roles in ovarian carcinogenesis [8–10]. Overexpression of TMEM88 in ovarian cancer is linked to platinum resistance [11]. TMEM119, also known as Osteoblast Induction Factor (OBIF), plays important role in bone formation and normal bone mineralization [12] and its overexpression enhances viability, invasion and migration of gastric cancer cells [13]. Associated with unfavorable prognosis in osteosarcoma patients, it’s found to stimulate osteosarcoma cell migration and invasion through TGF-β/BMP signaling [14]. Shorter overall survival has been observed in prostate cancer patients with upregulated TMEM119 expression [15]. However, its functions in ovarian cancer still need to be investigated.

In the present study, we firstly reported the oncogenic roles for TMEM119 in ovarian cancer by detecting its clinical significance in ovarian cancer patients and exploring its influence on malignant behaviors of ovarian cancer cells. Mechanistically, we observed that it could upregulate the expression of platelet-derived growth factor receptor beta (PDGFRB) and activate PI3K/AKT signaling pathway, thereby promoting the proliferation, invasion, and migration of ovarian cancer cells.

## Methods

### Analyses of expression and prognosis for TMEM119 in ovarian cancer

TMEM119 mRNA expression in ovarian cancer and normal ovarian tissues was analyzed using the GEPIA database (http://gepia.cancer-pku.cn/) [16]. TMEM119 protein expression in ovarian cancer and normal ovarian tissues was determined from the Human Protein Atlas database (www.proteinatlas.org). Prognostic value of TMEM119 in ovarian cancer was investigated using the Kaplan–Meier plotter database (http://kmplot.com) [17] and the Human Protein Atlas database. GEPIA database was also used for correlation analyses between the mRNA expression level of TMEM119 and that of other genes.

### Patients and tissue samples

Ovarian primary cancer and pseudonormal ovarian tissues were obtained from patients who underwent resection in Shengjing Hospital of China Medical University from 2012 to 2015. The inclusion criteria for ovarian cancer patients were as follows: ovarian primary cancer patients whose pathological diagnosis was confirmed by the pathologists; ovarian primary cancer patients receiving standardized cytoreductive surgery and postoperative chemotherapy at Shengjing Hospital of China Medical University. The exclusion criteria were as follows: patients received chemotherapy before the surgery; patients with no contact information and no access to prognostic information. The pseudonormal ovarian tissues were obtained from patients with undesired fertility due to operations for cervical cancer in situ. Ovarian cancer patients were followed through August 30, 2018. The study was approved by the Research Ethics Committee of China Medical University, and all patients signed written informed consent.

### Gene set enrichment analysis, GSEA

The transcriptomic data of ovarian cancer samples was downloaded from the TCGA databases (https://portal.gdc.cancer.gov/projects/TCGA-OV). The transcriptomic data of 44 ovarian cancer cell lines in the Cancer Cell Line Encyclopedia (CCLE) project were obtained from the GEO database (accession numbers: GSE36133) (https://www.ncbi.nlm.nih.gov/geo/), uniformly processed, and normalized using the Robust Multichip Average algorithm with the Bioconductor package “affy” in the R programming language (version 3.5.2; http://www.r-project.org). GSEA software (version 4.3.0; https://www.gsea-msigdb.org/gsea/index.jsp) was used for GSEA analysis. The gene sets database was c2.cp.kegg.v7.1.symbols.gmt. Enrichment analysis was performed on the expression spectrum data and attribute files after high and low grouping using the default-weighted enrichment analysis method. The random assortment frequency was set as 1,000.

### Cell culture, siRNA transfection, and lentiviral infection

A2780 and OVCAR-3 cells were purchased from the Chinese Academy of Sciences Cell Bank (Shanghai, China), were cultured at 37 °C in a 5% CO2 atmosphere, maintained in RPMI 1640 medium (Bioind, Kibbutz Beit Haemek, Israel) and supplemented with 10% fetal bovine serum (FBS; Bioind, Kibbutz Beit Haemek, Israel).

Small interfering RNAs (siRNAs) targeting TMEM119 and PDGFRB were ordered from GenePharma (Suzhou, China). Lipofectamine 3000 (Invitrogen, Carlsbad, USA) was used for transfection. The sequences for each siRNA are shown in Additional file 1. The lentiviral TMEM119 vector and the negative control vector were purchased from Genechem (Shanghai, China). The A2780 and OVCAR-3 cells were infected with lentiviral particles at the multiplicity of infection (MOI) of 10 and 30 for 48 h, respectively.

### Cell proliferation assay

Cell proliferation was investigated using a BeyoClick™EdU-488 cell proliferation kit (Beyotime Biotechnology, China). After incubation with EdU working solution (10 μM) for 2 h, cells were fixed by the addition of 4% paraformaldehyde for 15 min at room temperature. Cells were then incubated with 0.3% Triton X-100 in PBS for 10 min. Finally, cells were incubated with click reaction solution for 30 min in the dark. Images were captured by using a fluorescence microscope (Nikon, Japan) and were analyzed by ImageJ (https://imagej.en.softonic.com).

Cell viability was evaluated using a CCK-8 kit (Bimake, Houston, USA). Cell suspension was inoculated in 96-well plates (3 × 10^3 ^cells/well). CCK-8 solution (10 μl) was added to the wells every 24 h and the cells were incubated for 1.5 h. The absorbance was measured at 450 nm using a microplate reader.

### Transwell assay

Matrigel invasion assays were performed in 24-well plates using Transwell polycarbonate filters with an 8.0-μm pore size (Corning, New York, USA). 600 μl medium containing 10% FBS was added to the lower chamber. Cells in serum-free medium (2 × 10^4 ^cells/200 μl) were seeded into the upper chambers pre-coated with Matrigel (BD Biosciences, San Jose, USA). After incubation at 37 °C for 24 h, the cells were fixed with 4% paraformaldehyde and stained with 0.5% crystal violet. The invaded cells were photographed and counted.

### Cell scratch assay

Single cell suspensions were seeded in 6-well plates. The plate was gently scratched in a straight line with a 200 μL pipette tip and washed with PBS, then cultured in medium without serum for 24 h. Images were captured by microscope (Nikon, Japan) at 0 h and 24 h, and were analyzed by ImageJ (https://imagej.en.softonic.com).

### Reverse transcription quantitative polymerase chain reaction

Total tissue RNA was extracted using the RNAiso Plus reagent (Takara Bio, Kusatsu, Japan). RNA concentration and purity were assessed with a Nano Drop 2000 system (Thermo Fisher, Carlsbad, USA). Total RNA was reverse transcribed using the PrimeScript™ RT reagent Kit with gDNA Eraser (Perfect Real Time) (Takara Bio, Kusatsu, Japan). Real‐time quantitative polymerase chain reaction (qPCR) was performed using SYBR® Premix Ex Taq™ II (Tli RNaseH Plus) (Takara Bio, Kusatsu, Japan). The primer sequences were shown in Additional file 1. The PCR reactions were performed on an ABI 7500 Fast system (Life Technologies, Carlsbad, USA). Gene expression was calculated relative to that of ACTB using the 2 − △△Ct method.

### Western blot

Total cell or tissue proteins were extracted using RIPA lysate (Beyotime, Shanghai, China). Proteins were separated using 10% SDS‐PAGE and transferred onto PVDF membranes (Millipore, Massachusetts, USA). After being blocked at room temperature for 2 h, the membranes were incubated with primary anti-TMEM119 (1:1000; Proteintech, Chicago, USA), anti-PDGFRB (1:1000; Proteintech, Chicago, USA), anti-PI3K p85 (1:1000; Cell Signaling Technology, Danvers, USA), anti-p-PI3K p85 (1:1000; Cell Signaling Technology, Danvers, USA), anti-AKT (1:1000; Cell Signaling Technology, Danvers, USA), anti-p-AKT (1:1000; Cell Signaling Technology, Danvers, USA), anti-mTOR (1:1000; Cell Signaling Technology, Danvers, USA), anti-p-mTOR (Ser2448) (1:1000; Cell Signaling Technology, Danvers, USA) or anti-GAPDH (1:3000; Proteintech, Chicago, USA) antibodies. The membranes were then washed and incubated with secondary antibodies. Protein bands were detected with enhanced chemiluminescence (Thermo Scientific, Carlsbad, USA).

### Statistical analysis

Statistical analyses were performed with GraphPad Prism 7.0. Data were expressed as the mean ± standard deviation of at least three independent experiments. The count data were analyzed by Chi squared tests. The measurement data were analyzed using t-test. Univariable survival analysis was performed using the Kaplan–Meier method and the log-rank test. Multivariable survival analysis was performed using the multivariate Cox regression method. *P* < 0.05 was considered statistically significant. *P < 0.05; **P < 0.01; ***P < 0.001.

## Results

### TMEM119 is overexpressed in ovarian cancer and involved in ovarian cancer progression

**Fig. 1:**
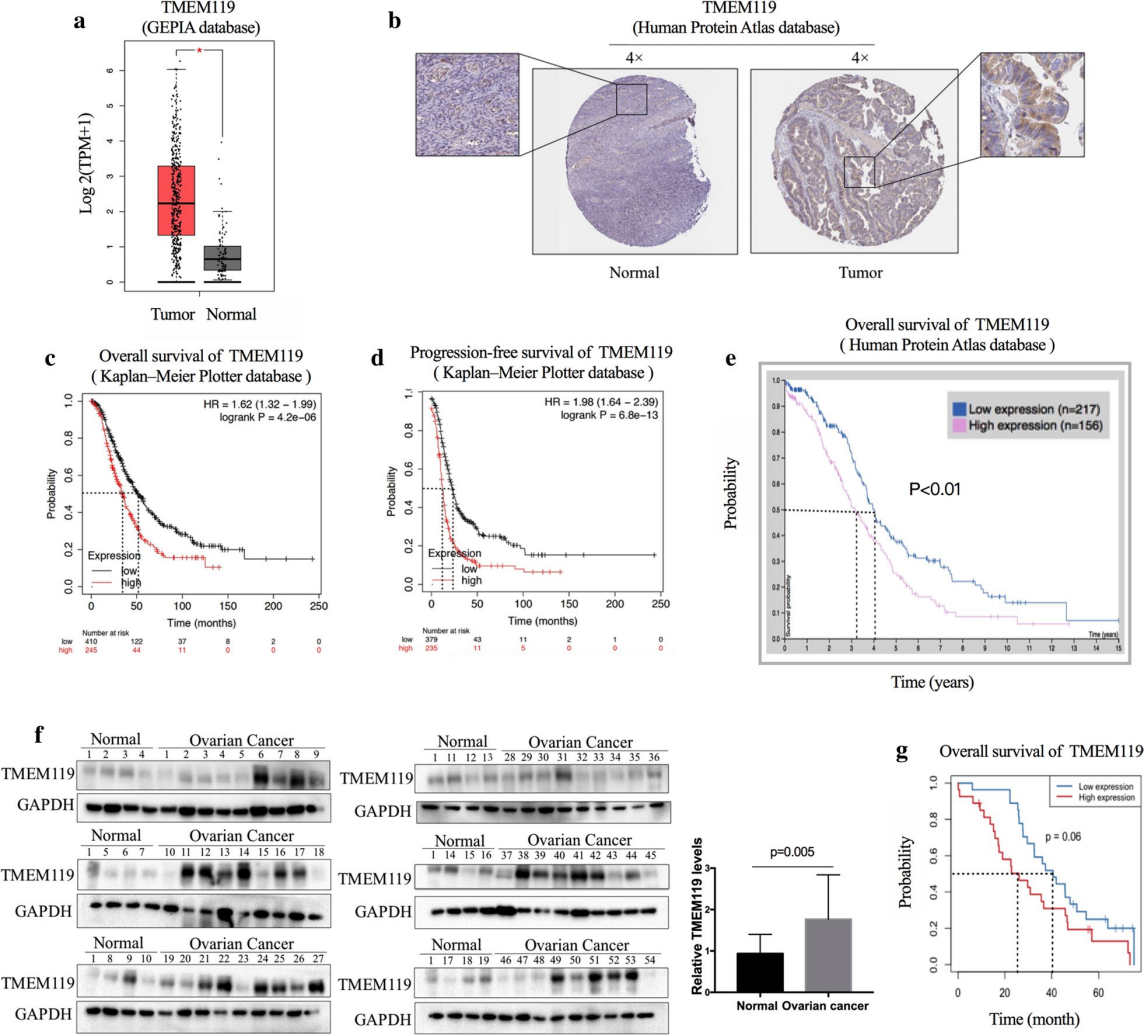
Expression levels and prognostic values of TMEM119 in ovarian cancer. **a** mRNA expression level of TMEM119 in ovarian cancer (426 samples) and normal ovarian tissues (88 samples) (GEPIA). **b** TMEM119 protein expression in ovarian cancer and normal ovarian tissue (Human Protein Atlas database). **c**, **d** Prognostic values of TMEM119 in ovarian cancer (Kaplan–Meier Plotter). The median overall survivals for the high- and low-expression groups are 33.5 and 50.97 months, respectively. The median progression-free survivals for the high- and low-expression groups are 12 and 22.6 months, respectively. **e** Prognostic value of TMEM119 in ovarian cancer (Human Protein Atlas database). **f** Expression levels of TMEM119 in clinical samples (54 ovarian cancer and 19 pseudonormal ovarian tissues). **g** Prognostic significance of TMEM119 in ovarian cancer patients (n = 54). The median overall survivals for the high- and low-expression groups are 26.1 and 40.5 months, respectively

To investigate the functions of TMEM119 in ovarian cancer, we firstly examined the expression level and prognostic value of TMEM119 in various database. In GEPIA database, higher mRNA level of TMEM119 was detected in ovarian cancer tissues compared to that in normal ovarian tissues (Fig. 1a). Consistently, we observed positive expression of TMEM119 in ovarian cancer tissue and negative expression in normal ovarian tissue in the Human Protein Atlas database (Fig. 1b). The mRNA expression of TMEM119 was found to be negatively associated with overall and progression-free survival in the Kaplan–Meier plotter database (Fig. 1c, d). High TMEM119 protein expression also induced poor survival in ovarian cancer patients in the Human Protein Atlas database (Fig. 1e).

Furthermore, we explored the expression of TMEM119 protein in 54 primary epithelial ovarian cancer specimen and 19 pseudonormal ovarian tissues via Western blot and observed higher TMEM119 protein levels in cancer tissues (Fig. 1f). Fifty-four patients with epithelial ovarian cancer were divided into high TMEM119-expression group and low TMEM119-expression group with the median protein expression level as the cutoff value. Kaplan–Meier analysis indicated that high TMEM119 expression was associated with a shortened overall survival (Fig. 1g). The average survival time in the low TMEM119-expression group was 42.27 months, while that in the high TMEM119-expression group was 29.36 months. However, after including known variables associated with prognosis (Additional file 2), TMEM119 expression level did not turn out to be an independent prognostic factor in ovarian cancer. Statistical analyses showed that TMEM119 expression level was positively correlated with International Federation of Gynecology and Obstetrics (FIGO) stage (Table 1). Therefore, TMEM119 could be a gene involved in ovarian cancer progression.

**Table 1:** Relationships between TMEM119 expression in epithelial ovarian cancer and clinicopathological parameters

Characteristic	n	Low	High	*P* value
Stage				0.038
FIGO I	6	5	1	
FIGO II	5	3	2	
FIGO III	34	18	16	
FIGO IV	9	1	8	
Grade				0.129
Well/Moderate	15	10	5	
Poor	39	17	22	
Pathologic type				0.388
Serous	42	21	21	
Mucinous	3	2	1	
Endometrioid	2	2	0	
Clear cell carcinoma	2	1	1	
Others	5	1	4	

*FIGO* International Federation of Gynecology and Obstetrics

### TMEM119 knockdown inhibits proliferation, invasion and migration of ovarian cancer cells

**Fig. 2:**
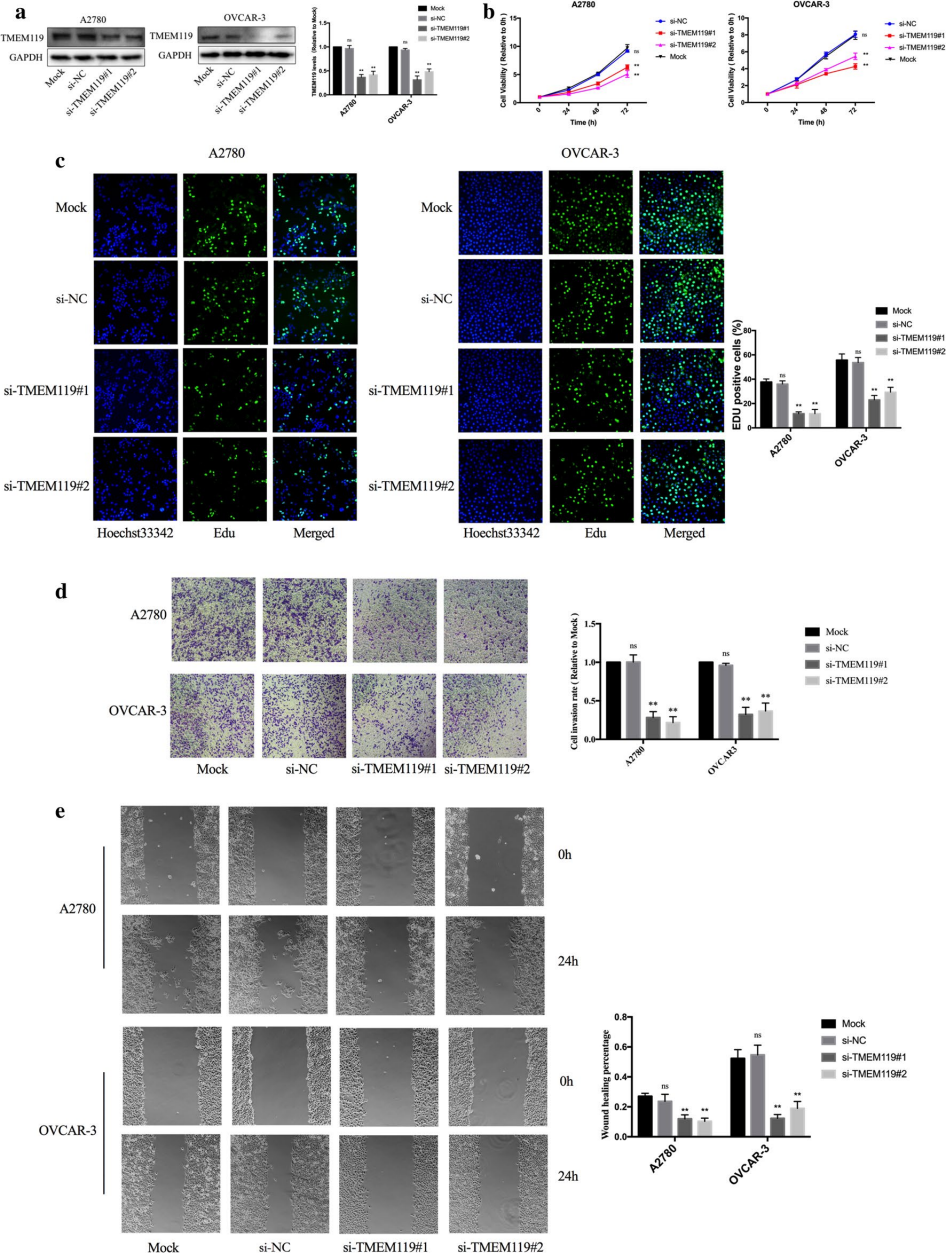
Effects of TMEM119 knockdown on the functions of ovarian cancer cell lines. **a** Efficiency of TMEM119 knockdown examined by Western blot. **b** Cell viability decreased after TMEM119 knockdown in the A2780 and OVCAR-3 cell lines. **c** Percentage of EdU positive cells decreased after TMEM119 knockdown in the A2780 and OVCAR-3 cell lines. **d** Cell invasion decreased after TMEM119 knockdown in the A2780 and OVCAR-3 cell lines. **e** Wound healing percentage decreased after TMEM119 knockdown in the A2780 and OVCAR-3 cell lines

To further explore the effects of TMEM119 on the proliferation, invasion, and migration of ovarian cancer cells, we downregulated TMEM119 expression in OVCAR-3 and A2780 cells using RNA interference. The efficiency of knockdown was shown in Fig. 2a. CCK-8 experiments indicated that under-expressed TMEM119 attenuated cell viability (Fig. 2b). EdU experiments demonstrated that the proliferative ability of ovarian cancer cells decreased after knockdown of TMEM119 (Fig. 2c). Via Transwell and scratch assays, invasion and migration abilities of cells were demonstrated to be significantly reduced after TMEM119 knockdown (Fig. 2d, e).

### TMEM119 overexpression promotes proliferation, invasion and migration of ovarian cancer cells

**Fig. 3:**
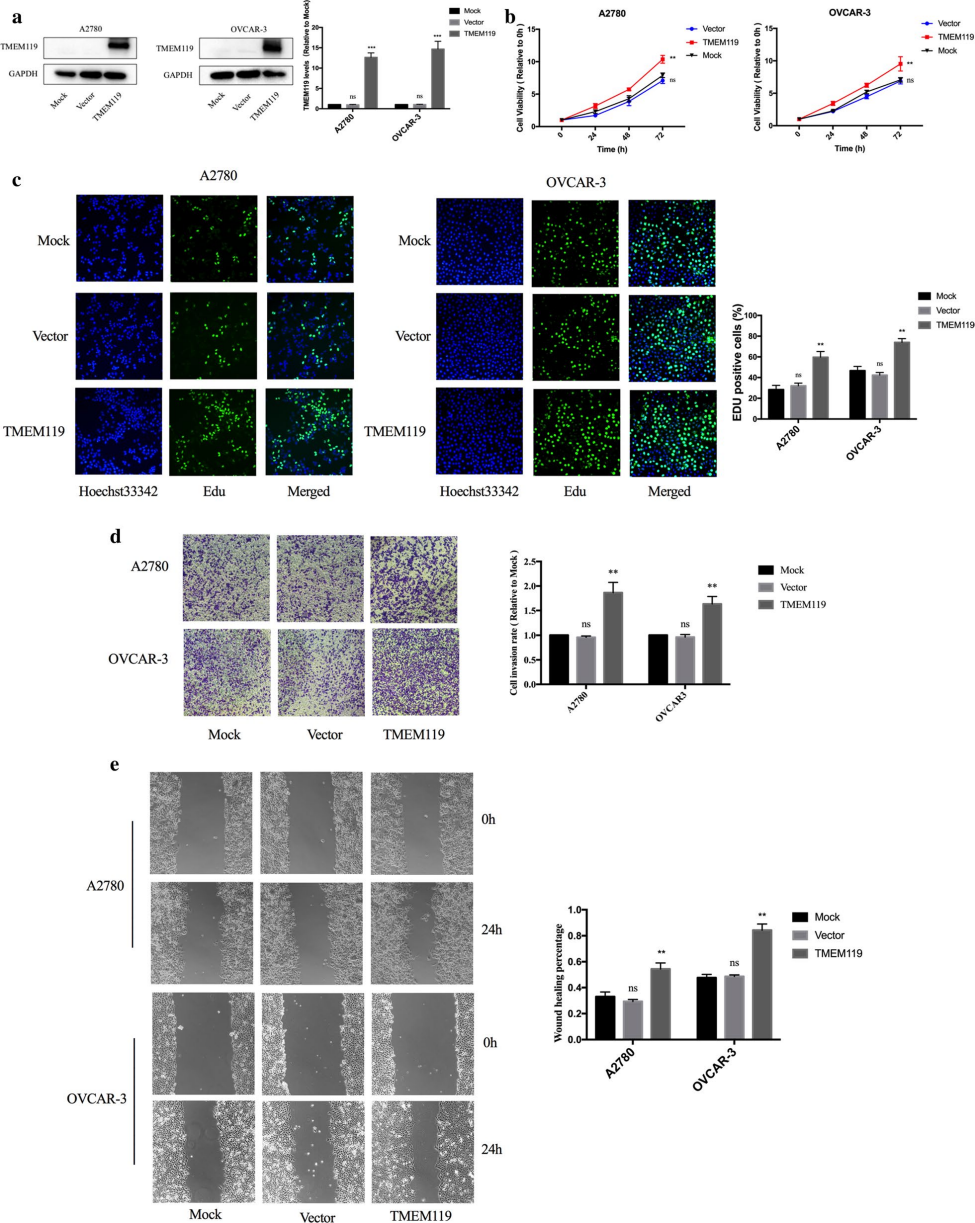
Effects of TMEM119 overexpression on the functions of ovarian cancer cell lines. **a** Efficiency of TMEM119 overexpression examined by Western blot. **b** Cell viability increased after TMEM119 overexpression in the A2780 and OVCAR-3 cell lines. **c** Percentage of EdU positive cells increased after TMEM119 overexpression in the A2780 and OVCAR-3 cell lines. **d** Cell invasion increased after TMEM119 overexpression in the A2780 and OVCAR-3 cell lines. **e** Wound healing percentage increased after TMEM119 overexpression in the A2780 and OVCAR-3 cell lines

We then established OVCAR-3 and A2780 cell lines stably overexpressing TMEM119 by lentiviral infection. The efficiency of overexpression was shown in Fig. 3a. CCK-8 experiments demonstrated that over-expressed TMEM119 induced an increase in cell viability of ovarian cancer cells (Fig. 3b). Enhanced proliferative ability was also shown in TMEM119 over-expressed cells according to the results of the EdU experiment (Fig. 3c). In Transwell and scratch assays, the invasion and migration abilities of cells elevated after TMEM119 overexpression (Fig. 3d, e). Therefore, the expression of TMEM119 was positively associated with proliferation, invasion and migration of ovarian cancer cells.

### Pathway enrichment analysis of TMEM119

**Fig. 4:**
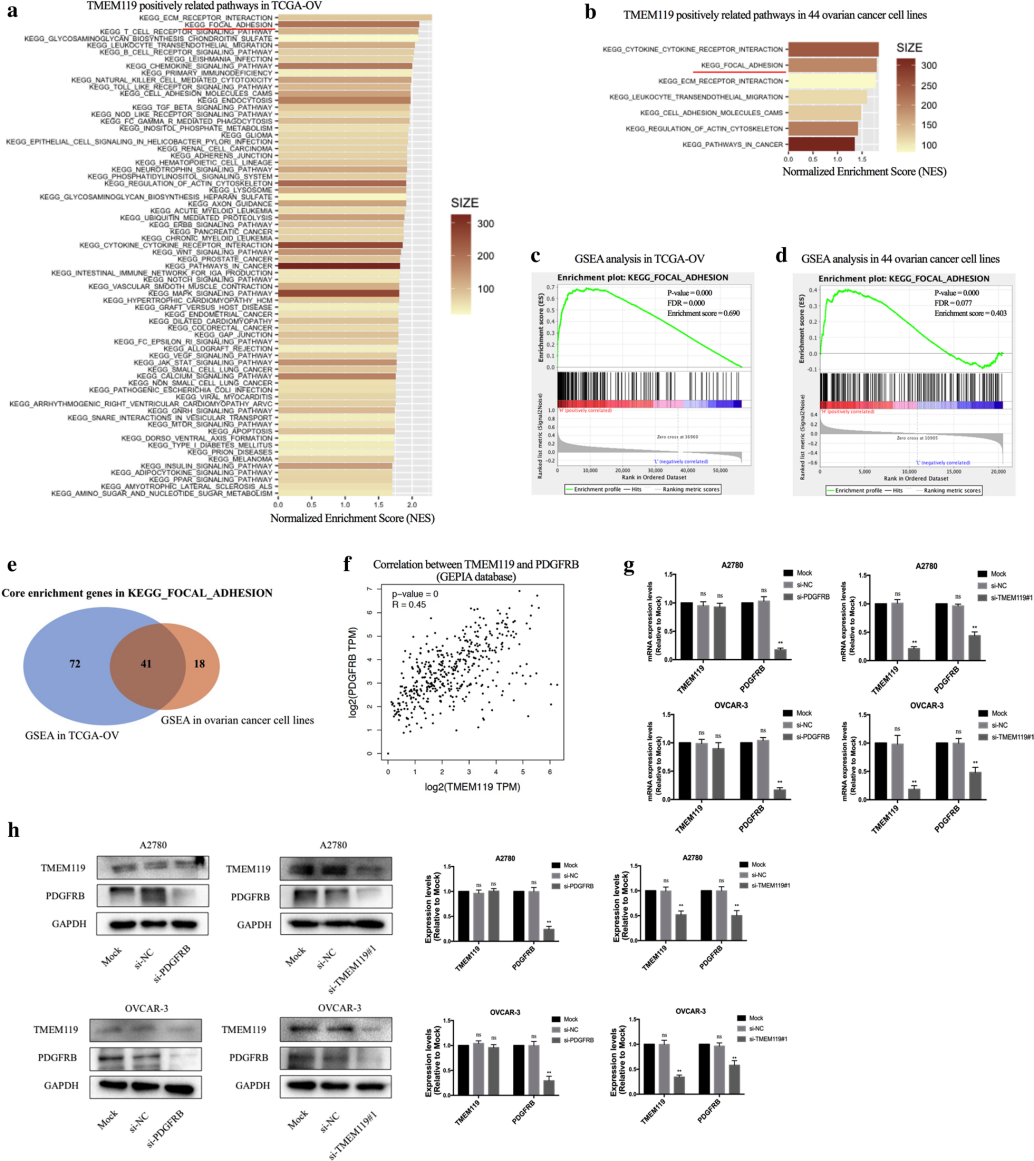
Pathway enrichment analysis of TMEM119. **a**, **b** Pathway associated with TMEM119 in GSEA analyses based on the transcriptomic data of OV patients from the TCGA database (only pathway with NOM p-value < 0.001 and FDR q-value < 0.001 was shown) and the transcriptomic data of 44 ovarian cancer cell lines from the CCLE project (only pathway with NOM p-value < 0.01 was shown) **c**–**d** KEGG_FOCAL_ADHESION pathway in GSEA analyses based on the transcriptomic data of OV patients from the TCGA database and the transcriptomic data of 44 ovarian cancer cell lines from the CCLE project. **e** Venn diagram for the screening of the core enrichment genes in KEGG_FOCAL_ADHESION pathway in both GSEA analyses.** f** Correlation analyses between the mRNA expression level of TMEM119 and that of PDGFRB in GEPIA database.** g**–**h** mRNA and protein expression levels of TMEM119 and PDGFRB in ovarian cancer cells after knockdown of TMEM119 or PDGFRB

To further understand the molecular mechanisms of TMEM119 in progression of ovarian cancer, we conducted GSEA analyses in the transcriptomic data of OV patients from the TCGA database (Fig. 4a) and in the transcriptomic data of 44 ovarian cancer cell lines from the CCLE project (Fig. 4b), respectively. The median value of TMEM119 mRNA level was set as a cut-off point for distinguishing high expression group and low expression group in each dataset. As indicated by GSEA analyses in the two datasets, TMEM119 expression was strongly associated with the KEGG FOCAL ADHESION pathway (Fig. 4c, d), which was found to be associated with various biological processes including cell motility, cell proliferation, cell differentiation, regulation of gene expression and cell survival [18]. In this pathway, we identified 113 core enrichment genes according to GSEA analysis in the transcriptomic data of OV patients (Additional file 3) and 59 core enrichment genes according to GSEA analysis in the transcriptomic data of ovarian cancer cell lines (Additional file 4). Then, a Venn diagram was generated to explore the intersection of these genes (Fig. 4e). Forty-one genes were recognized as core enrichment genes in KEGG FOCAL ADHESION pathway of both GSEA analyses. We used GEPIA database for correlation analysis between mRNA expression level of TMEM119 and mRNA expression level of the 41 genes and identified PDGFRB as the most correlated gene with TMEM119 (Fig. 4f).

Previous study has disclosed PDGFRB as a gene involved in cancer progression [19]. We then analyzed the expression of PDGFRB protein and found that its levels were higher in ovarian cancer tissues (Additional file 5). Furthermore, statistical analyses indicated that PDGFRB expression wasn’t an independent prognostic factor in ovarian cancer but was positively associated with FIGO stage (Additional files 5, 6, 7). Therefore, PDGFRB is associated with ovarian cancer progression. In order to further detect the relationship between TMEM119 and PDGFRB in ovarian cancer progression, we downregulated PDGFRB expression by RNA interference and found there was no significant change in both mRNA and protein expression level of TMEM119 (Fig. 4g, h). Nevertheless, the expression of PDGFRB was inhibited after downregulation of TMEM119, indicating that TMEM119 can regulate the expression of PDGFRB.

### TMEM119 exerts oncogenic effects by regulating PDGFRB

**Fig. 5:**
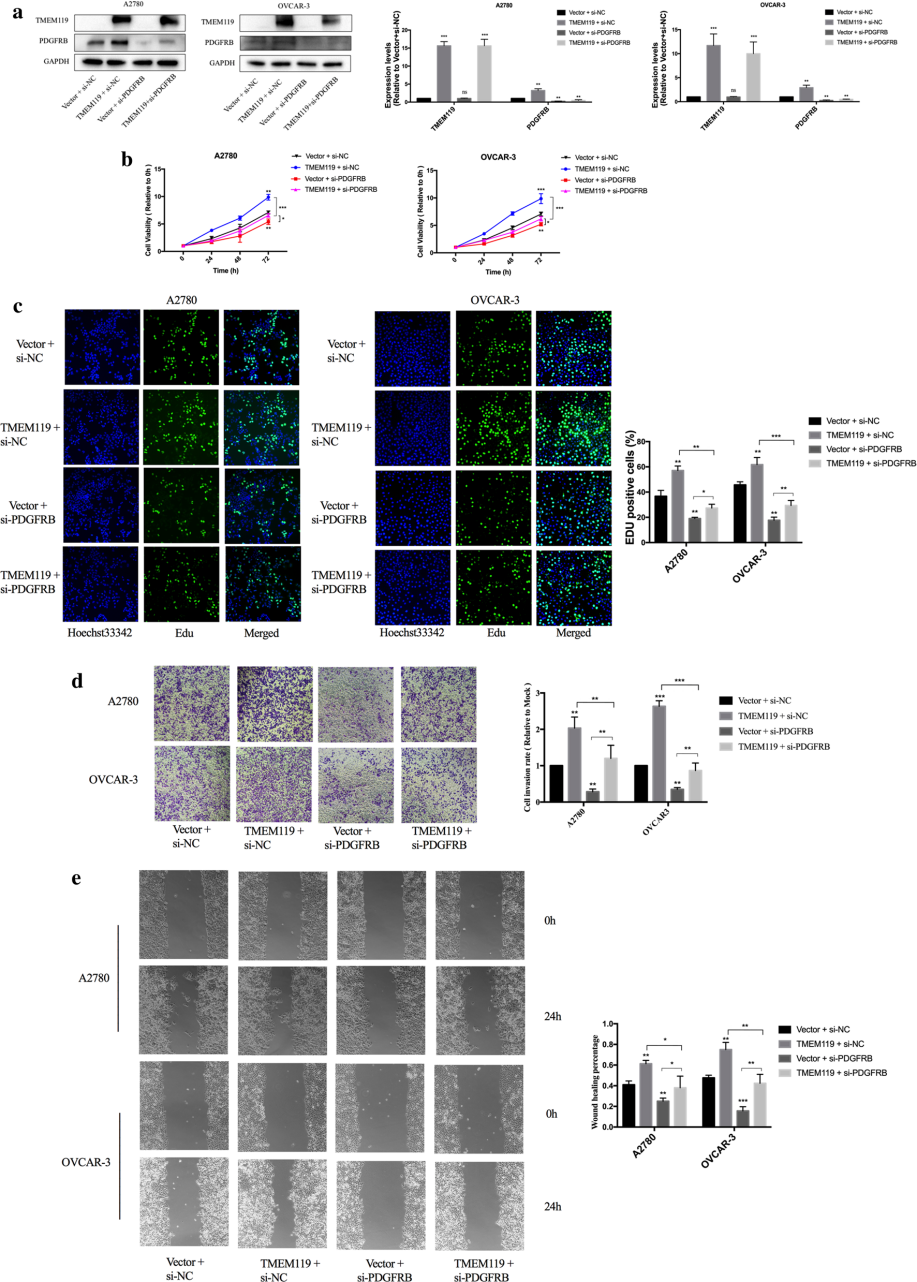
Effects of PDGFRB knockdown on the functions of ovarian cancer cell lines. **a** Efficiency of PDGFRB knockdown examined by Western blot. **b** Cell viability decreased after PDGFRB knockdown in ovarian cancer cell lines. PDGFRB knockdown partially abolished the promotion of viability induced by TMEM119 overexpression. **c** Percentage of EdU positive cells decreased after PDGFRB knockdown in ovarian cancer cell lines. PDGFRB knockdown partially abolished the promotion of proliferation induced by TMEM119 overexpression. **d** Cell invasion decreased after PDGFRB knockdown in ovarian cancer cell lines. PDGFRB knockdown partially abolished the promotion of invasion induced by TMEM119 overexpression. **e** Wound healing percentage decreased after PDGFRB knockdown in ovarian cancer cell lines. PDGFRB knockdown partially abolished the promotion of migration induced by TMEM119 overexpression

To explore whether PDGFRB is implicated in TMEM119-induced proliferation, migration and invasion of ovarian cancer cells, PDGFRB expression was downregulated by using RNA interference in ovarian cancer cells. The knockdown efficacy was validated in Fig. 5a. As shown in Fig. 5b, c, CCK-8 and EdU experiments demonstrated that knockdown of PDGFRB significantly attenuated viability and proliferation of ovarian cancer cells. Downregulation of PDGFRB also inhibited invasive and migratory abilities of ovarian cancer cells (Fig. 5d, e). Moreover, we inhibited the expression of PDGFRB in TMEM119-overexpressed cells. Downregulation of PDGFRB expression partially abolished the promotion of viability, proliferation, invasion and migration induced by TMEM119 overexpression (Fig. 5b–e). We also utilized GZD856, a PDGFRA and PDGFRB inhibitor which suppresses proliferation of lung cancer [20], and observed that GZD856 effectively inhibit proliferation, invasion and migration of ovarian cancer cells (Additional file 8). Additionally, TMEM119-overexpressed cells had decreased sensitivity to GZD856 (Additional file 8). Taken together, these findings suggested that TMEM119 can function an upstream of PDGFRB to regulate ovarian cancer progression.

### TMEM119 overexpression activates the PI3K/AKT signaling pathway

**Fig. 6:**
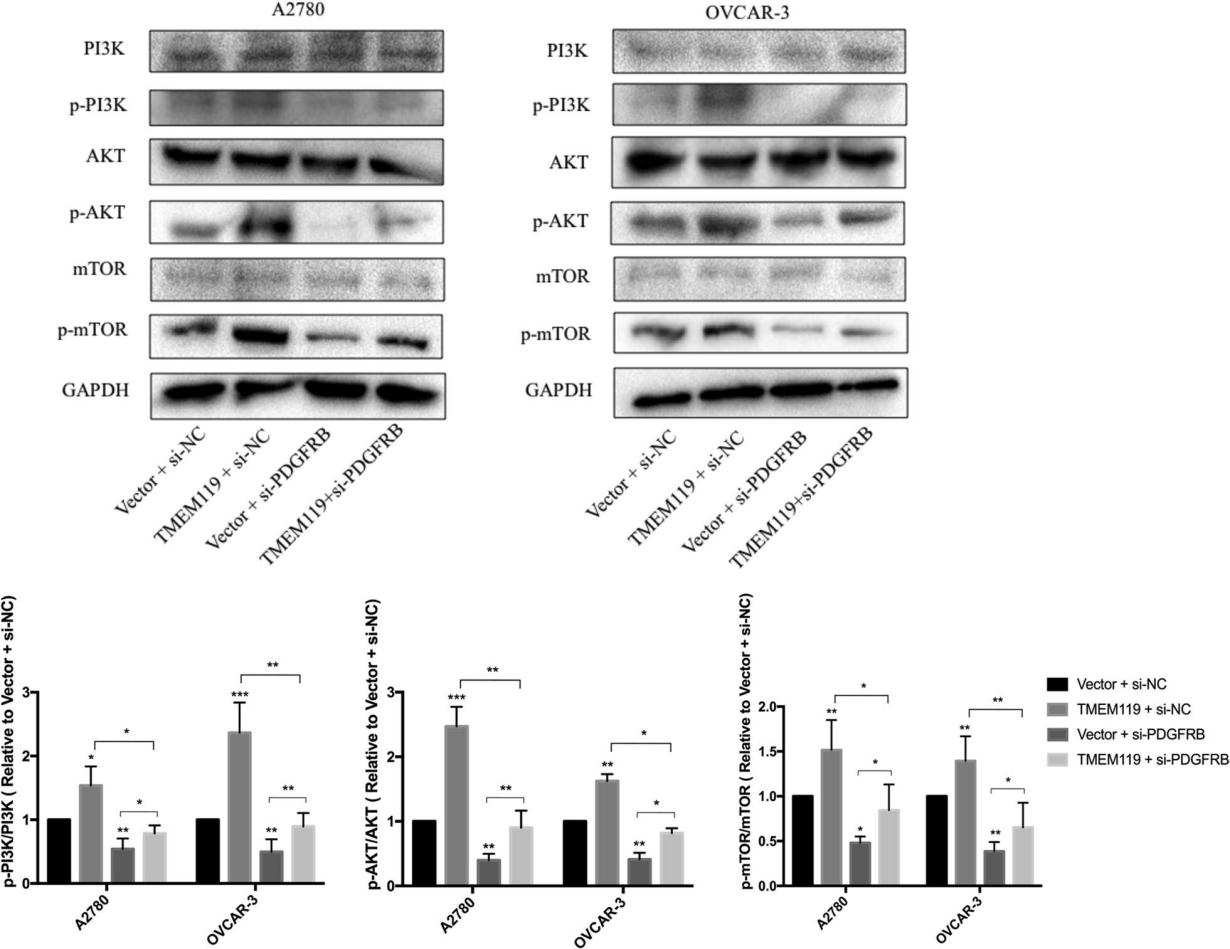
Effects of TMEM119 overexpression and PDGFRB knockdown on the PI3K-AKT signaling pathways in the A2780 and OVCAR-3 cell lines

Playing important roles in the pathway of KEGG FOCAL ADHESION [18], the PI3K/AKT signaling pathway is a tyrosine kinase cascade pathway and is connected with the proliferation and progression of ovarian cancer [21]. Thus, we further explored the effects of TMEM119 on the PI3K/AKT signaling by performing western blot analyses. Phosphorylation of both PI3K, AKT and their important downstream signaling molecule mTOR elevated significantly after TMEM119 overexpression (Fig. 6). The downregulation of PDGFRB expression could partially abolished the increased phosphorylation of PI3K and AKT induced by TMEM119 overexpression. These findings suggest that TMEM119 exerts oncogenic effects by activating the PI3K/AKT signaling pathway.

## Discussion

Multiple TMEM family members, such as TMEM45a and TMEM49, have been reported to contribute to progression of ovarian cancer [8–11], highlighting the potential importance of this family in tumorigenesis of ovarian cancer. TMEM119 was firstly identified in 2009 to be associated with osteoblast differentiation via BMP2-RUNX214 and ATF4/RUNX2/Osterix signaling pathways [12]. In recent years, more and more studies have revealed the involvement of TMEM119 in various cancers, including gastric cancer, osteosarcoma, and hepatocellular carcinoma [13, 14, 22]. However, the function of TMEM119 in ovarian cancer is still unclear. In the present study, we analyzed the role of TMEM119 in ovarian cancer for the first time by combining various datasets and clinical samples, demonstrating that TMEM119 was upregulated in ovarian cancer tissues and was positively associated with FIGO stage, which indicated that TMEM119 could be a gene involved in ovarian cancer progression.

In order to investigate the effect of TMEM119 on the malignant behavior of ovarian cancer cells, we respectively carried out functional experiments after knockdown and overexpression of TMEM119. Our results revealed that TMEM119 promoted proliferation, invasion and migration of ovarian cancer cells in vitro. TMEM119 has been reported by previous study to enhance gastric cancer cell migration and invasion by activating STAT3 signaling pathway [23]. Its silence can inhibit viability and promote apoptosis of gastric cancer cells [13]. Study also found that TMEM119 promotes proliferation, migration and invasion of osteosarcoma cells partly through TGF-β/BMP signaling [14]. Taken together, TMEM119 is disclosed to act as an oncogenic factor by stimulating malignant behaviors of multiple cancer cells.

To further explore the molecular mechanisms of TMEM119 involved in ovarian cancer progression, we combined datasets from TCGA and CCLE databases to investigate the pathways associated with TMEM119 in ovarian cancer. Results of GSEA analyses demonstrated that TMEM119 gene was significantly related to the pathways of ECM receptor interaction, focal adhesion, cytokine cytokine receptor interaction and leukocyte transendothelial migration (Fig. 4a, b), indicating that TMEM119 participate in transmitting information between cell and the extracellular environment. We focused on pathway of focal adhesion because of its known association with various carcinogenesis processes [18] and identified PDGFRB as the downstream of TMEM119. PDGFRB is a tyrosine-protein kinase that acts as cell-surface receptor for homodimeric PDGFB and PDGFD and for heterodimers formed by PDGFA and PDGFB [24, 25]. Studies have reported that PDGFRB plays important roles in the regulation of cell proliferation, survival, differentiation, chemotaxis and migration [26, 27]. Activation of PDGFRB/ERK pathway has been found to promote progression of triple-negative breast cancer [28]. PDGFRB has been also disclosed to promote cancer stem cell phenotypes and epithelial-mesenchymal transition in sarcomas [19]. PDGFR was previously demonstrated to be a potential target in epithelial ovarian cancer [29]. Studies have reported that PDGFRB is expressed in a high percentage of epithelial ovarian cancers [30, 31]. Our results indicated that PDGFRB was upregulated in ovarian cancer and positively associated with FIGO stage (Additional file 5, 6). Knockdown of PDGFRB could inhibit proliferation, invasion and migration of ovarian cancer cells in vitro, disclosing that PDGFRB was involved in ovarian cancer progression. We also uncovered PDGFRB inhibitor GZD856 as a potential drug in ovarian cancer treatment. Moreover, TMEM119 was revealed to be a regulator of PDGFRB expression. TMEM119 promoted proliferation, invasion and migration of ovarian cancer cells partially via upregulating PDGFRB. Accruing evidence suggests that TMEMs contribute to cancer development by acting as receptors, channels, anchorage proteins or structural proteins [32]. Nevertheless, the detailed mechanism by which TMEM119 participates in carcinogenesis and cancer progression remains elusive. Previous studies observed that TMEM119 protein mediates cell–cell interactions and cell–cell communication in testis differentiation [33]. During the process of osteoblastic differentiation, TMEM119 has been reported to be complexed with multiple transcription factors, including Smad1, Smad5 and Runx2 [34]. In recent years, TMEM119 has been regarded as a microglial receptor and a marker of microglial cells [35]. Some cancer associated signaling, such as TGF-β pathway, could be altered by TMEM119 [14]. Therefore, it still requires further exploration to figure out whether TMEM119 upregulates the transcription of PDGFRB in ovarian cancer by interacting directly with transcription factors and by binding with ligands to transfer signal into the cytoplasm or by other potential mechanisms.

PI3K/AKT signaling pathway plays a key role in the regulation of various cellular functions, including metabolism, growth, proliferation, transcription, and protein synthesis [36]. Abnormal activation of this pathway is involved in tumorigenesis and progression of various cancers, including ovarian cancer [21, 37, 38]. Inhibitors of this pathway has been used for targeted therapy against ovarian cancer [39, 40]. Our study demonstrated that PI3K/AKT signaling could be activated by TMEM119 in ovarian cancer cells. The activation was partly dependent on PDGFRB. Consistently, previous studies have clarified that PDGFRB could activate PI3K/AKT signaling by phosphorylation of PIK3R1, inducing proliferation and migration of cells [41, 42]. Thus, these observations suggested that TMEM119 could regulate PDGFRB and PI3K/AKT signaling pathway, affecting proliferation, invasion, and migration in ovarian cancer.

## Conclusions

In conclusion, the present study demonstrated that TMEM119 is overexpressed in ovarian cancer and promotes ovarian cancer progression. TMEM119 can regulate proliferation, invasion, and migration in ovarian cancer cells via the PDGFRB/PI3K/AKT signaling pathway. These findings provided a rationale for further developing therapeutics by targeting TMEM119 in ovarian cancer.

## Supplementary Information

**Additional file 1.** The sequences for siRNAs and primers.

**Additional file 2. **Multivariable Cox regression analysis for TMEM119.

**Additional file 3. **The core enrichment genes of KEGG FOCAL ADHESION pathway according to GSEA analysis in the transcriptomic data of OV patients.

**Additional file 4.** The core enrichment genes of KEGG FOCAL ADHESION pathway according to GSEA analysis in the transcriptomic data of ovarian cancer cell lines.

**Additional file 5.** Expression levels and prognostic values of PDGFRB in ovarian cancer. a Expression levels of PDGFRB in clinical samples (54 ovarian cancer and 19 pseudonormal ovarian tissues). b Prognostic significance of PDGFRB in ovarian cancer patients (n=54). c-d Prognostic values of PDGFRB in ovarian cancer (Kaplan–Meier Plotter). The median overall survivals for the high- and low-expression groups are 40.1 and 49.5 months, respectively. The median progression-free survivals for the high- and low-expression groups are 18.2 and 23 months, respectively.

**Additional file 6. **Relationships between PDGFRB expression in epithelial ovarian cancer and clinicopathological parameters.

**Additional file 7.** Multivariable Cox regression analysis for PDGFRB.

**Additional file 8. **Effects of GZD856 on the functions of ovarian cancer cell lines. a Cell viability decreased after utilization of GZD856 in the A2780 and OVCAR-3 cell lines. b Percentage of EdU positive cells decreased after utilization of GZD856 for 48h in the A2780 and OVCAR-3 cell lines. c Cell invasion decreased after utilization of GZD856 for 24 h in the A2780 and OVCAR-3 cell lines. d Wound healing percentage decreased after utilization of GZD856 for 24h in the A2780 and OVCAR-3 cell lines.

## Data Availability

Not applicable.

## Abbreviations

OV: Ovarian cancer
TMEM119: Transmembrane proteins 119
PDGFRB: Platelet-derived growth factor receptor beta
OBIF: Osteoblast induction factor
CCLE: Cancer Cell Line Encyclopedia
FIGO: Federation of Gynecology and Obstetrics
