# Supplementary material for: TMEM119 facilitates ovarian cancer cell proliferation, invasion, and migration via the PDGFRB/PI3K/AKT signaling pathway
Source: J Transl Med. 2021 Mar 17;19:111. doi: 10.1186/s12967-021-02781-x (PMC7968362; doi:10.1186/s12967-021-02781-x)
Supplement: Supplementary file 1 — Additional file 1. The sequences for siRNAs and primers. [file 12967_2021_2781_MOESM1_ESM.pdf]

The sequences for siRNA

Control siRNA	Forward, UUCUCCGAAC GUGUCACGUT T
	Reverse, ACGUGACACG UUCGGAGAAT T
TMEM119 siRNA#1	Forward, CCUAUUACCC AUCGUCCUUT T
	Reverse, AAGGACGAUG GGUAAUAGG TT
TMEM119 siRNA#2	Forward, GCCUCCUCAU CCUUCUGUUT T
	Reverse, AACAGAAGGA UGAGGAGGCT T
PDGFRB siRNA	Forward, GAGGGUGACA ACGACUAUAT T
	Reverse, UAUAGUCGUU GUCACCCUCT T

The sequences for primer

TMEM119	Forward, CGGCCTATTA CCCATCGTCC
	Reverse, CTGGGCTAAC AAGAGAGACC C
PDGFRB	Forward, CGGGAATGAG GTGGTCAACT TCG
	Reverse, AGGATGGAGC GGATGTGGTA AGG
