# Supplementary material for: TMEM119 facilitates ovarian cancer cell proliferation, invasion, and migration via the PDGFRB/PI3K/AKT signaling pathway
Source: J Transl Med. 2021 Mar 17;19:111. doi: 10.1186/s12967-021-02781-x (PMC7968362; doi:10.1186/s12967-021-02781-x)
Supplement: Supplementary file 2 — Additional file 2. Multivariable Cox regression analysis for TMEM119. [file 12967_2021_2781_MOESM2_ESM.pdf]

Table 1. Multivariable Cox regression analysis for TMEM119

Characteristic	OR	95%CI	P value
Stage			
FIGO I / II	1 (reference)		
FIGO III / IV	3.512	1.290, 9.563	0.014
Grade			
Well/Moderate	1 (reference)		
Poor	0.677	0.310, 1.481	0.329
Pathologic type			
Serous	1 (reference)		
Mucinous	2.723	0.796, 9.317	0.111
Endometrioid	1.163	0.143, 9.430	0.888
Clear cell carcinoma	3.245	0.662, 15.908	0.147
Others	1.413	0.484, 4.128	0.527
TMEM119			
Low	1 (reference)		
High	1.382	0.727, 2.627	0.324

FIGO, International Federation of Gynecology and Obstetrics
