# Supplementary material for: TMEM119 facilitates ovarian cancer cell proliferation, invasion, and migration via the PDGFRB/PI3K/AKT signaling pathway
Source: J Transl Med. 2021 Mar 17;19:111. doi: 10.1186/s12967-021-02781-x (PMC7968362; doi:10.1186/s12967-021-02781-x)
Supplement: Supplementary file 3 — Additional file 3. The core enrichment genes of KEGG FOCAL ADHESION pathway according to GSEA analysis in the transcriptomic data of OV patients. [file 12967_2021_2781_MOESM3_ESM.pdf]

NAME	PROBE	RANK IN GENE LIST	RANK METRIC SCORE	RUNNING ES	CORE ENRICHMENT
row_0	COL3A1	4	0.567449331	0.01595883	Yes
row_1	COL5A2	6	0.556539893	0.031662747	Yes
row_2	COL1A1	7	0.555569232	0.047356997	Yes
row_3	COL1A2	9	0.545857608	0.06275915	Yes
row_4	PDGFRB	12	0.536913276	0.07789088	Yes
row_5	COL6A3	14	0.533265233	0.09293731	Yes
row_6	ITGA11	24	0.522608221	0.10754065	Yes
row_7	COL5A1	38	0.51061362	0.121734135	Yes
row_8	COL11A1	39	0.509147406	0.13611701	Yes
row_9	FN1	44	0.502162457	0.15023157	Yes
row_10	THBS2	47	0.499047786	0.16429363	Yes
row_11	COL6A2	55	0.489958555	0.17801017	Yes
row_12	ITGA4	75	0.467874467	0.19088978	Yes
row_13	ITGA5	77	0.465310574	0.20401657	Yes
row_14	PIK3R5	93	0.455363899	0.21661378	Yes
row_15	COL6A1	100	0.449586421	0.2292076	Yes
row_16	COL4A1	119	0.440470546	0.24133083	Yes
row_17	COL4A2	173	0.421142429	0.25228664	Yes
row_18	COL5A3	207	0.408388317	0.26323727	Yes
row_19	COL6A6	298	0.387061536	0.27257335	Yes
row_20	THBS1	301	0.385922641	0.28343976	Yes
row_21	TNN	319	0.383036613	0.2939583	Yes
row_22	PIK3CG	360	0.374745876	0.30383426	Yes
row_23	ITGA1	487	0.343700618	0.31130627	Yes
row_24	SHC1	511	0.339542925	0.32048965	Yes
row_25	LAMB1	531	0.336128682	0.32964757	Yes
row_26	TLN1	537	0.335113227	0.3390254	Yes
row_27	PIK3CD	596	0.325428039	0.3471886	Yes
row_28	ITGB1	671	0.314620793	0.3547624	Yes
row_29	CRKL	706	0.310134798	0.3629197	Yes
row_30	MAPK1	736	0.307156056	0.37108162	Yes
row_31	PARVA	889	0.289495587	0.37656078	Yes
row_32	VASP	901	0.288457692	0.3845141	Yes
row_33	RAPGEF1	939	0.284907371	0.3919055	Yes
row_34	FLNC	972	0.282197297	0.39930907	Yes
row_35	ACTB	986	0.281757176	0.40703762	Yes
row_36	KDR	999	0.280883074	0.4147592	Yes
row_37	PDGFB	1084	0.27446726	0.42102116	Yes
row_38	ITGB5	1110	0.27317661	0.42829424	Yes
row_39	LAMA4	1114	0.272872806	0.43594933	Yes
row_40	FLT1	1131	0.271996349	0.44334885	Yes
row_41	ARHGAP35	1223	0.265166134	0.4492238	Yes
row_42	RAC2	1232	0.263898849	0.45653662	Yes
row_43	VCL	1242	0.26320374	0.46381205	Yes
row_44	ACTN1	1293	0.260425687	0.47028103	Yes
row_45	PIP5K1C	1332	0.258216977	0.4769007	Yes
row_46	PGF	1377	0.256033421	0.48335212	Yes

row_47	FLNA	1419	0.253918707	0.48979712	Yes
row_48	ILK	1487	0.249696597	0.49566117	Yes
row_49	PDGFRA	1508	0.248951569	0.50233865	Yes
row_50	DOCK1	1608	0.243624866	0.50746304	Yes
row_51	MYLK	1669	0.241469711	0.513219	Yes
row_52	THBS3	1812	0.235426426	0.5173483	Yes
row_53	TNC	1816	0.235342696	0.5239432	Yes
row_54	PDGFC	1896	0.232539922	0.52910954	Yes
row_55	CTNNB1	1948	0.230770379	0.53472304	Yes
row_56	ITGAV	1959	0.23057808	0.5410591	Yes
row_57	AKT2	2012	0.22785604	0.5465725	Yes
row_58	VAV2	2200	0.222000837	0.54952353	Yes
row_59	PRKCA	2287	0.219121873	0.5541865	Yes
row_60	PIK3CA	2341	0.217614129	0.55939287	Yes
row_61	PXN	2344	0.217582658	0.56550384	Yes
row_62	IBSP	2406	0.216101795	0.5705254	Yes
row_63	LAMC1	2536	0.212546095	0.5742392	Yes
row_64	SPP1	2561	0.211738735	0.57979447	Yes
row_65	GSK3B	2620	0.210298985	0.58470535	Yes
row_66	PDGFD	2624	0.210151076	0.5905886	Yes
row_67	BCAR1	2629	0.20990698	0.5964473	Yes
row_68	GRB2	3005	0.201042101	0.5954682	Yes
row_69	PDPK1	3051	0.199937865	0.6003173	Yes
row_70	CHAD	3091	0.199015573	0.60524684	Yes
row_71	SRC	3106	0.198736593	0.61061233	Yes
row_72	SOS1	3131	0.197975785	0.6157788	Yes
row_73	ROCK2	3133	0.19796294	0.6213533	Yes
row_74	ITGB7	3135	0.197956383	0.6269276	Yes
row_75	PAK2	3177	0.197367698	0.6317751	Yes
row_76	PTEN	3213	0.196549937	0.636706	Yes
row_77	PPP1R12A	3380	0.193321869	0.6392197	Yes
row_78	PARVG	3467	0.191620395	0.64310586	Yes
row_79	HGF	3587	0.18943055	0.6463442	Yes
row_80	ITGA8	3637	0.188663617	0.6508037	Yes
row_81	LAMA5	4096	0.180699915	0.6477763	Yes
row_82	ZYX	4275	0.178006023	0.6496444	Yes
row_83	COMP	4449	0.175127834	0.65151983	Yes
row_84	ITGB6	4600	0.172655895	0.6537339	Yes
row_85	RAF1	4695	0.171406657	0.65690696	Yes
row_86	VWF	4703	0.171292245	0.6616215	Yes
row_87	ROCK1	4722	0.170986325	0.6661321	Yes
row_88	MAPK8	4837	0.169717565	0.66890234	Yes
row_89	IGF1	5119	0.1653139	0.66858304	Yes
row_90	MYL9	5443	0.161621734	0.66741365	Yes
row_91	CCND3	5447	0.16160135	0.6719255	Yes
row_92	EGFR	5595	0.159850642	0.67383105	Yes
row_93	CDC42	5748	0.157976955	0.6755949	Yes
row_94	MAPK9	5809	0.157273725	0.6789724	Yes

row_95	PRKCB	5906	0.156176835	0.6816797	Yes
row_96	SHC3	6452	0.149900123	0.6762376	Yes
row_97	VAV1	6999	0.144353122	0.670621	Yes
row_98	ACTN2	7399	0.140573218	0.66750765	Yes
row_99	ARHGAP5	7696	0.138023362	0.66615105	Yes
row_100	ACTN4	7900	0.136246607	0.66639555	Yes
row_101	RAP1A	7976	0.135599121	0.6688944	Yes
row_102	IGF1R	8223	0.133576751	0.66830003	Yes
row_103	PAK4	8294	0.132909	0.67081165	Yes
row_104	BRAF	8387	0.132289842	0.6729152	Yes
row_105	ITGB8	8483	0.13145715	0.674942	Yes
row_106	PIK3CB	8503	0.131266385	0.6783128	Yes
row_107	CRK	8675	0.129910052	0.67894644	Yes
row_108	MYLK2	8806	0.128805339	0.6802769	Yes
row_109	LAMB2	8807	0.128801644	0.6839154	Yes
row_110	RAP1B	8870	0.12840122	0.6864417	Yes
row_111	TLN2	8881	0.128358185	0.68989015	Yes
row_112	ITGA10	9074	0.126900956	0.690066	Yes
row_113	ACTG1	9555	0.123402625	0.6850294	No
row_114	PARVB	9594	0.123103306	0.68783224	No
row_115	DIAPH1	10099	0.119601823	0.6822621	No
row_116	XIAP	10454	0.116997898	0.67928183	No
row_117	MYL10	10923	0.114252731	0.6741998	No
row_118	JUN	11165	0.112682723	0.6731039	No
row_119	MAPK3	11242	0.112151831	0.6749227	No
row_120	RHOA	11281	0.111972585	0.67741114	No
row_121	ITGA2	11331	0.111694746	0.6796964	No
row_122	ITGA3	11571	0.110273957	0.67856795	No
row_123	ACTN3	11597	0.110081472	0.68123376	No
row_124	PPP1CC	11653	0.109743953	0.68335736	No
row_125	MAP2K1	11985	0.107521094	0.6805177	No
row_126	RAC1	12136	0.106603719	0.6808658	No
row_127	SOS2	12292	0.105624817	0.68109757	No
row_128	PAK3	12371	0.105305627	0.6826874	No
row_129	LAMB3	13105	0.101083599	0.6725282	No
row_130	VEGFC	13239	0.10023611	0.6729983	No
row_131	MYL12A	13353	0.099632099	0.6738065	No
row_132	BIRC2	13739	0.097527228	0.6697257	No
row_133	AKT3	13758	0.097454563	0.6721591	No
row_134	CAPN2	13951	0.096411817	0.67147356	No
row_135	VEGFD	14251	0.094741583	0.66884106	No
row_136	CCND1	14259	0.094723672	0.6713926	No
row_137	COL4A6	14308	0.094397455	0.673207	No
row_138	ITGB3	14448	0.093705676	0.6733861	No
row_139	COL11A2	14628	0.092725463	0.6728273	No
row_140	MAPK10	15289	0.089514911	0.66363746	No
row_141	PPP1CA	16374	0.084240697	0.64677036	No
row_142	FYN	17881	0.077551328	0.6222215	No

row_143	PTK2	18421	0.074938439	0.6147683	No
row_144	VEGFB	18696	0.073735602	0.6119863	No
row_145	ITGA2B	18778	0.073546998	0.6126257	No
row_146	CAV1	19654	0.069765665	0.5990606	No
row_147	COL4A4	19798	0.06926024	0.5984781	No
row_148	SHC4	20824	0.064837091	0.5821104	No
row_149	PDGFA	21913	0.060551718	0.56450313	No
row_150	FLNB	22866	0.056942072	0.5492086	No
row_151	VEGFA	22920	0.056630712	0.5498673	No
row_152	COL2A1	23099	0.055922844	0.5482866	No
row_153	ITGA9	23268	0.055158313	0.5468618	No
row_154	LAMC2	23574	0.053907469	0.5429693	No
row_155	ITGA7	24161	0.051704418	0.53402525	No
row_156	RAC3	24287	0.051146187	0.53325063	No
row_157	THBS4	25634	0.045645621	0.5106414	No
row_158	ITGB4	26539	0.042211019	0.49578294	No
row_159	BIRC3	26655	0.041687395	0.4949187	No
row_160	PPP1CB	27087	0.039807189	0.48839065	No
row_161	VTN	27450	0.038102742	0.48303956	No
row_162	MYL7	27963	0.036276355	0.4749736	No
row_163	CAV3	28443	0.034717124	0.4674495	No
row_164	LAMA3	28531	0.034386095	0.46687615	No
row_165	LAMA2	29248	0.031448543	0.4550517	No
row_166	HRAS	30126	0.028320713	0.44028026	No
row_167	TNXB	30527	0.02666999	0.4339315	No
row_168	CCND2	30732	0.025810815	0.43103856	No
row_169	BAD	30825	0.025401419	0.4301226	No
row_170	BCL2	30939	0.024997897	0.42882243	No
row_171	LAMC3	31186	0.024110153	0.4251357	No
row_172	CAV2	33420	0.016361894	0.38595018	No
row_173	MET	33874	0.014404912	0.3783139	No
row_174	AKT1	33876	0.014403104	0.37870303	No
row_175	ELK1	34629	0.011224605	0.36566806	No
row_176	PAK6	34639	0.011170149	0.3658238	No
row_177	TNR	34645	0.01114172	0.3660498	No
row_178	LAMA1	34702	0.010881368	0.36536288	No
row_179	PIK3R1	34950	0.009812741	0.36125448	No
row_180	LAMB4	36015	0.0050622	0.34250578	No
row_181	PAK1	36037	0.004980664	0.34227362	No
row_182	SHC2	40639	-0.006430136	0.26076284	No
row_183	MYL12B	40799	-0.007183504	0.25814265	No
row_184	FLT4	41190	-0.009093756	0.25147495	No
row_185	MYL5	42357	-0.01465595	0.23118621	No
row_186	PAK5	44167	-0.022124359	0.19969174	No
row_187	RELN	44439	-0.023689788	0.19554925	No
row_188	ERBB2	45716	-0.030212019	0.17374685	No
row_189	ITGA6	45776	-0.030555902	0.17356245	No
row_190	PIK3R3	46603	-0.034294546	0.1598653	No

row_191	VAV3	47837	-0.040847406	0.13912684	No
row_192	EGF	48945	-0.047412939	0.120811015	No
row_193	MYLK3	49268	-0.049580991	0.1164944	No
row_194	PRKCG	50852	-0.059626296	0.090072036	No
row_195	MYL2	51779	-0.066631697	0.07551284	No
row_196	MYLPF	53348	-0.080285564	0.04994041	No
row_197	RASGRF1	55065	-0.105442323	0.022450838	No
row_198	PIK3R2	55598	-0.118508682	0.01635273	No
