# Supplementary material for: TMEM119 facilitates ovarian cancer cell proliferation, invasion, and migration via the PDGFRB/PI3K/AKT signaling pathway
Source: J Transl Med. 2021 Mar 17;19:111. doi: 10.1186/s12967-021-02781-x (PMC7968362; doi:10.1186/s12967-021-02781-x)
Supplement: Supplementary file 4 — Additional file 4. The core enrichment genes of KEGG FOCAL ADHESION pathway according to GSEA analysis in the transcriptomic data of ovarian cancer cell lines. [file 12967_2021_2781_MOESM4_ESM.pdf]

NAME	PROBE	RANK IN GENE LIST	RANK METRIC SCORE	RUNNING ES	CORE ENRICHMENT
row_0	COL6A3	84	0.267549366	0.014428957	Yes
row_1	COL4A1	92	0.263752252	0.032389354	Yes
row_2	FLNC	100	0.25605607	0.049815606	Yes
row_3	COL4A2	137	0.24382785	0.0649639	Yes
row_4	LAMA1	152	0.238534927	0.08082912	Yes
row_5	FN1	200	0.221113458	0.09385881	Yes
row_6	LAMB1	220	0.215075493	0.107849434	Yes
row_7	ACTN3	238	0.210735902	0.12163744	Yes
row_8	COL1A1	373	0.179468125	0.12748897	Yes
row_9	CAV2	390	0.177557796	0.13902357	Yes
row_10	KDR	403	0.176222682	0.15066266	Yes
row_11	ITGA10	410	0.175148904	0.16252294	Yes
row_12	PDGFD	416	0.174752414	0.174405	Yes
row_13	LAMC1	453	0.168790981	0.18434544	Yes
row_14	COL6A1	470	0.166617081	0.19512072	Yes
row_15	RAC3	510	0.162097543	0.20444877	Yes
row_16	PDGFC	570	0.155348495	0.2123227	Yes
row_17	SHC4	591	0.154082119	0.22203088	Yes
row_18	COL6A2	597	0.15373759	0.2324544	Yes
row_19	ITGB3	610	0.152674213	0.24245915	Yes
row_20	SPP1	614	0.152418047	0.25288966	Yes
row_21	ITGA5	618	0.151984781	0.26329014	Yes
row_22	MYLK	623	0.151433483	0.27360305	Yes
row_23	MYL12A	662	0.148776636	0.28205585	Yes
row_24	TNC	663	0.148636922	0.2923718	Yes
row_25	CAV1	692	0.146169379	0.30113652	Yes
row_26	VEGFA	696	0.145932183	0.3111169	Yes
row_27	MYL7	728	0.142946959	0.3195101	Yes
row_28	TLN2	766	0.140067756	0.32740778	Yes
row_29	LAMB3	778	0.138933361	0.33650815	Yes
row_30	VCL	841	0.134216607	0.3427676	Yes
row_31	VWF	1271	0.112246163	0.32941446	Yes
row_32	PDGFRB	1379	0.107462712	0.33159924	Yes
row_33	ITGAV	1425	0.106134884	0.33674756	Yes
row_34	BCAR1	1447	0.105470203	0.3430326	Yes
row_35	RAC2	1497	0.103250317	0.34778357	Yes
row_36	ZYX	1500	0.103101172	0.3548406	Yes
row_37	PAK3	1510	0.102881759	0.36153743	Yes
row_38	PIK3CB	1582	0.100695044	0.36502677	Yes
row_39	COL11A2	1665	0.098105989	0.3677943	Yes
row_40	FLNA	1670	0.097885616	0.37439078	Yes
row_41	PARVB	1810	0.093858398	0.37405425	Yes
row_42	LAMB2	1910	0.091054007	0.37549448	Yes
row_43	TNN	1950	0.090083994	0.37982452	Yes
row_44	EGFR	1986	0.089383528	0.3843031	Yes
row_45	MAPK8	2025	0.08854758	0.38857576	Yes
row_46	ACTN1	2109	0.086563155	0.3904929	Yes

row_47	ITGA1	2133	0.085945323	0.39532426	Yes
row_48	BIRC3	2242	0.083748743	0.3958139	Yes
row_49	CCND1	2444	0.079310142	0.391412	Yes
row_50	PRKCB	2551	0.07740707	0.39156008	Yes
row_51	CAPN2	2628	0.07606601	0.39309365	Yes
row_52	PAK4	2683	0.074934192	0.39563295	Yes
row_53	PIK3CD	2780	0.073319457	0.3959902	Yes
row_54	MYL10	2851	0.072206192	0.39755163	Yes
row_55	FLT1	2858	0.072142839	0.4022629	Yes
row_56	SRC	3100	0.068524659	0.39514098	Yes
row_57	ITGA8	3137	0.068055928	0.39809003	Yes
row_58	VAV1	3138	0.06805294	0.40281317	Yes
row_59	CHAD	3334	0.065363169	0.39773896	No
row_60	TLN1	3459	0.063762702	0.39605296	No
row_61	PGF	3703	0.060470603	0.3882735	No
row_62	ITGB1	3776	0.059588507	0.3888606	No
row_63	PDGFRA	3858	0.058641009	0.38893843	No
row_64	LAMA5	3894	0.0582086	0.39125332	No
row_65	AKT2	4018	0.056930948	0.38914245	No
row_66	VEGFC	4260	0.054077011	0.3810178	No
row_67	FLNB	4420	0.052349202	0.37681466	No
row_68	IGF1R	4432	0.052271262	0.37990037	No
row_69	LAMB4	4451	0.052050162	0.3826257	No
row_70	ITGB7	4906	0.047519721	0.3635482	No
row_71	CRKL	5012	0.046282671	0.3615854	No
row_72	ITGA2B	5125	0.045102865	0.35919577	No
row_73	TNR	5184	0.04453611	0.3594282	No
row_74	ILK	5273	0.043761387	0.35812828	No
row_75	BIRC2	5275	0.043729499	0.361114	No
row_76	CAV3	5353	0.043042019	0.3603063	No
row_77	PRKCA	5419	0.042442087	0.36004838	No
row_78	SOS1	5435	0.042322896	0.36224645	No
row_79	MYL9	5677	0.04021455	0.35315973	No
row_80	VAV3	5870	0.038415909	0.34636316	No
row_81	COL5A3	5900	0.038065169	0.34757575	No
row_82	ERBB2	6100	0.036352344	0.34029093	No
row_83	BCL2	6124	0.036079254	0.34166142	No
row_84	COL6A6	6253	0.034990575	0.33778137	No
row_85	ACTN4	6301	0.034660645	0.33787054	No
row_86	COL2A1	6520	0.032897092	0.3294095	No
row_87	ELK1	6542	0.032701485	0.33064413	No
row_88	MYL2	6682	0.031413119	0.32597363	No
row_89	TNXB	6710	0.031136965	0.32680395	No
row_90	MET	6754	0.030824501	0.32682404	No
row_91	CCND2	6943	0.029267449	0.31958964	No
row_92	ACTN2	6944	0.029265406	0.3216208	No
row_93	PDGFB	7198	0.027285336	0.3110453	No
row_94	LAMA2	7374	0.025884036	0.3042168	No

row_95	PRKCG	7856	0.022051508	0.28204098	No
row_96	DIAPH1	8057	0.020611014	0.2736144	No
row_97	PAK1	8319	0.018713543	0.2620497	No
row_98	COL4A4	8384	0.018187234	0.2601577	No
row_99	MYLK2	8901	0.014465878	0.23573045	No
row_100	PPP1R12A	9145	0.012793939	0.22464205	No
row_101	SOS2	9438	0.010762726	0.2109977	No
row_102	PTEN	9471	0.01051535	0.21015038	No
row_103	MYLK3	9855	0.007678342	0.19180699	No
row_104	BRAF	9871	0.007608538	0.19159578	No
row_105	ITGA11	9890	0.007479304	0.19122773	No
row_106	PDGFA	9907	0.007382384	0.19095153	No
row_107	VAV2	10019	0.006590755	0.18593827	No
row_108	RASGRF1	10110	0.005923153	0.18191367	No
row_109	GRB2	10319	0.004282489	0.17195955	No
row_110	ROCK2	10381	0.003923088	0.16922541	No
row_111	BAD	10661	0.001860203	0.1556039	No
row_112	LAMA3	10903	1.57E-05	0.14372723	No
row_113	PIK3R5	10904	6.33E-06	0.14372766	No
row_114	MAP2K1	10976	-4.72E-04	0.14026119	No
row_115	HGF	11102	-0.001268751	0.13418858	No
row_116	ITGA3	11149	-0.001613649	0.13203345	No
row_117	RAF1	11322	-0.0029673	0.12376231	No
row_118	CDC42	11337	-0.003082134	0.123286225	No
row_119	VASP	11593	-0.004893398	0.11105808	No
row_120	AKT1	11632	-0.005217065	0.10954732	No
row_121	RAP1A	11651	-0.005332946	0.10903031	No
row_122	PIK3CG	11769	-0.006098858	0.103687204	No
row_123	MYL12B	11791	-0.006254175	0.10308627	No
row_124	MYLPF	11953	-0.007519281	0.095673196	No
row_125	DOCK1	12078	-0.008435213	0.09014725	No
row_126	PPP1CA	12163	-0.008954173	0.08662873	No
row_127	RELN	12333	-0.010215708	0.07900851	No
row_128	PIK3R2	12341	-0.010272052	0.07937644	No
row_129	PPP1CC	12457	-0.011050487	0.074475564	No
row_130	PPP1CB	12516	-0.01153254	0.072417416	No
row_131	RHOA	12648	-0.012634926	0.066837944	No
row_132	PTK2	12661	-0.012816982	0.067136064	No
row_133	RAP1B	12777	-0.013682634	0.062417872	No
row_134	THBS2	12836	-0.014113631	0.060538862	No
row_135	FLT4	13027	-0.015836615	0.05227376	No
row_136	PIK3R1	13159	-0.016856873	0.04698731	No
row_137	MAPK1	13293	-0.017905299	0.041675054	No
row_138	LAMC3	13344	-0.01837839	0.040486313	No
row_139	IBSP	13621	-0.02100262	0.028341215	No
row_140	ACTB	13706	-0.021733301	0.025709616	No
row_141	LAMC2	13951	-0.024034707	0.015352087	No
row_142	JUN	14056	-0.024971524	0.011959527	No

row_143	ITGA2	14087	-0.025258953	0.012234031	No
row_144	VEGFB	14567	-0.029954029	-0.009294735	No
row_145	PARVA	14642	-0.030735873	-0.010808667	No
row_146	CTNNB1	14644	-0.030753922	-0.008723515	No
row_147	HRAS	14653	-0.030820047	-0.006978771	No
row_148	MAPK9	14750	-0.031883765	-0.009497313	No
row_149	PDPK1	14934	-0.034104057	-0.016149584	No
row_150	CRK	15342	-0.038956873	-0.033504974	No
row_151	COL11A1	15377	-0.039348815	-0.032449722	No
row_152	COL1A2	15429	-0.039927438	-0.03219216	No
row_153	PARVG	15470	-0.040412318	-0.03135881	No
row_154	ROCK1	15526	-0.040914379	-0.031229893	No
row_155	ARHGAP5	15631	-0.041987497	-0.03344148	No
row_156	SHC1	15867	-0.044757374	-0.041917212	No
row_157	CCND3	16115	-0.048177857	-0.050746974	No
row_158	LAMA4	16352	-0.051915918	-0.058775157	No
row_159	PIK3CA	16384	-0.052292362	-0.05667372	No
row_160	PIP5K1C	16421	-0.052919228	-0.0547752	No
row_161	ARHGAP35	16575	-0.054805622	-0.058512144	No
row_162	ITGA4	16859	-0.059156109	-0.06835425	No
row_163	RAC1	16907	-0.05999437	-0.066506825	No
row_164	SHC2	16978	-0.061186705	-0.06571022	No
row_165	MAPK10	17086	-0.062971488	-0.06661329	No
row_166	ITGA9	17098	-0.063274398	-0.06276395	No
row_167	THBS4	17101	-0.063304231	-0.058468968	No
row_168	GSK3B	17225	-0.065892905	-0.059957854	No
row_169	MAPK3	17322	-0.067542389	-0.060001552	No
row_170	ITGA7	17367	-0.068493322	-0.057416417	No
row_171	RAPGEF1	17874	-0.079903126	-0.076809235	No
row_172	ITGB5	18217	-0.087859981	-0.08756702	No
row_173	XIAP	18293	-0.089966409	-0.08501941	No
row_174	COL5A1	18368	-0.092065483	-0.082276836	No
row_175	PXN	18437	-0.094060779	-0.07910007	No
row_176	COL3A1	18460	-0.09484648	-0.07360165	No
row_177	PIK3R3	18649	-0.101125352	-0.07584882	No
row_178	COL5A2	18718	-0.103462338	-0.072019555	No
row_179	THBS3	18931	-0.111414574	-0.07473546	No
row_180	PAK6	19008	-0.114610404	-0.07052676	No
row_181	IGF1	19149	-0.12193843	-0.06896372	No
row_182	MYL5	19156	-0.122118592	-0.060783945	No
row_183	COL4A6	19244	-0.126235321	-0.056310568	No
row_184	ITGB4	19396	-0.13442497	-0.05442306	No
row_185	COMP	19417	-0.135715887	-0.045989577	No
row_186	THBS1	19624	-0.14819172	-0.045857303	No
row_187	ITGB8	19672	-0.151559055	-0.037654947	No
row_188	PAK2	19704	-0.154624626	-0.028451266	No
row_189	SHC3	19818	-0.163523898	-0.022671342	No
row_190	FYN	19853	-0.167644575	-0.012711883	No

row_191	EGF	19914	-0.174491167	-0.003558664	No
row_192	ITGA6	20026	-0.188397691	0.004046166	No
row_193	AKT3	20362	-0.265515	0.005963305	No
