# Supplementary figures and images for: TMEM119 facilitates ovarian cancer cell proliferation, invasion, and migration via the PDGFRB/PI3K/AKT signaling pathway
Source: J Transl Med. 2021 Mar 17;19:111. doi: 10.1186/s12967-021-02781-x (PMC7968362; doi:10.1186/s12967-021-02781-x)

a

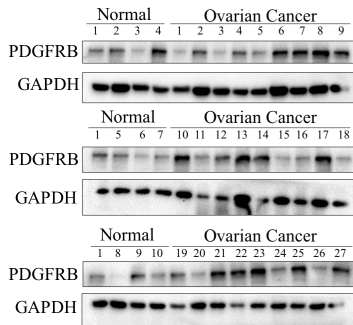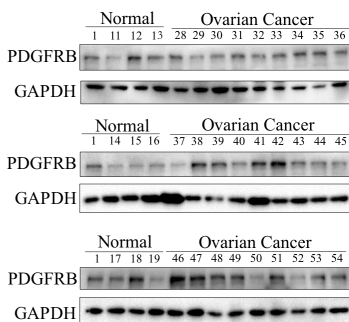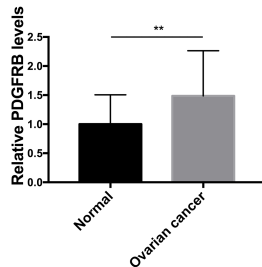

b

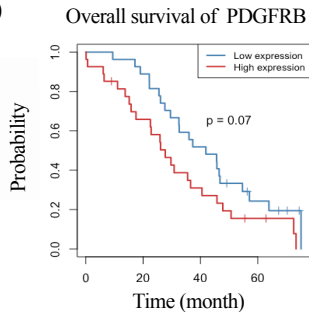

c

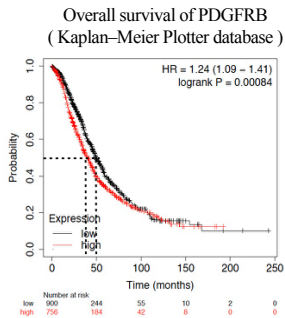

d

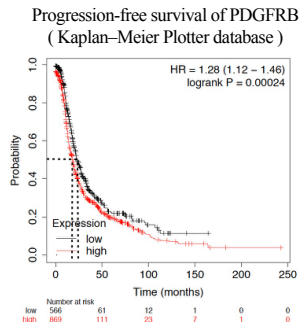

Supplement: Supplementary file 5 — Additional file 5. Expression levels and prognostic values of PDGFRB in ovarian cancer. a Expression levels of PDGFRB in clinical samples (54 ovarian cancer and 19 pseudonormal ovarian tissues). b Prognostic significance of PDGFRB in ovarian cancer patients (n=54). c-d Prognostic values of PDGFRB in ovarian cancer (Kaplan–Meier Plotter). The median overall survivals for the high- and low-expression groups are 40.1 and 49.5 months, respectively. The median progression-free survivals for the high- and low-expression groups are 18.2 and 23 months, respectively. [file 12967_2021_2781_MOESM5_ESM.pdf]

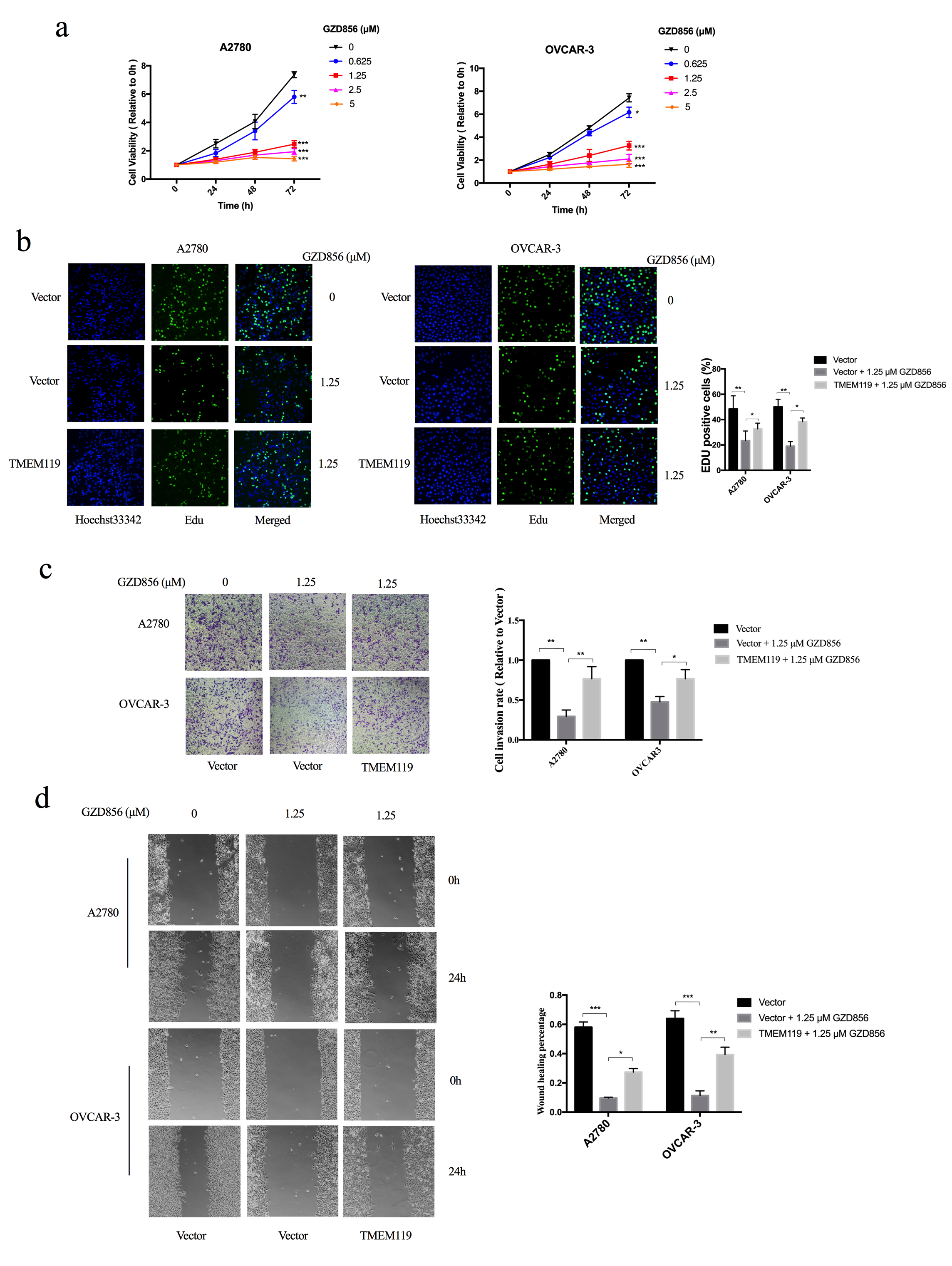

Supplement: Supplementary file 8 — Additional file 8. Effects of GZD856 on the functions of ovarian cancer cell lines. a Cell viability decreased after utilization of GZD856 in the A2780 and OVCAR-3 cell lines. b Percentage of EdU positive cells decreased after utilization of GZD856 for 48h in the A2780 and OVCAR-3 cell lines. c Cell invasion decreased after utilization of GZD856 for 24 h in the A2780 and OVCAR-3 cell lines. d Wound healing percentage decreased after utilization of GZD856 for 24h in the A2780 and OVCAR-3 cell lines. [file 12967_2021_2781_MOESM8_ESM.tiff]
