# Supplementary material for: TMEM119 facilitates ovarian cancer cell proliferation, invasion, and migration via the PDGFRB/PI3K/AKT signaling pathway
Source: J Transl Med. 2021 Mar 17;19:111. doi: 10.1186/s12967-021-02781-x (PMC7968362; doi:10.1186/s12967-021-02781-x)
Supplement: Supplementary file 6 — Additional file 6. Relationships between PDGFRB expression in epithelial ovarian cancer and clinicopathological parameters. [file 12967_2021_2781_MOESM6_ESM.pdf]

Table 1. Relationships between PDGFRB expression in epithelial ovarian cancer and clinicopathological parameters

Characteristic	n	Low	High	P value
Stage				0.018
FIGO I/II	11	9	2	
FIGO III/IV	43	18	25	
Grade				0.761
Well/Moderate	15	8	7	
Poor	39	19	20	
Pathologic type				0.572
Serous	42	23	19	
Mucinous	3	1	2	
Endometrioid	2	1	1	
Clear cell carcinoma	2	0	2	
Others	5	2	3	

FIGO, International Federation of Gynecology and Obstetrics
