# Supplementary material for: TMEM119 facilitates ovarian cancer cell proliferation, invasion, and migration via the PDGFRB/PI3K/AKT signaling pathway
Source: J Transl Med. 2021 Mar 17;19:111. doi: 10.1186/s12967-021-02781-x (PMC7968362; doi:10.1186/s12967-021-02781-x)
Supplement: Supplementary file 7 — Additional file 7. Multivariable Cox regression analysis for PDGFRB. [file 12967_2021_2781_MOESM7_ESM.pdf]

Table 1. Multivariable Cox regression analysis for PDGFRB

Characteristic	OR	95%CI	P value
Stage			
FIGO I / II	1 (reference)		
FIGO III / IV	3.611	1.303, 10.005	0.014
Grade			
Well/Moderate	1 (reference)		
Poor	0.655	0.293, 1.463	0.302
Pathologic type			
Serous	1 (reference)		
Mucinous	2.762	0.799, 9.548	0.109
Endometrioid	0.923	0.106, 8.011	0.942
Clear cell carcinoma	2.487	0.477, 12.959	0.279
Others	1.643	0.560, 4.821	0.366
PDGFRB			
Low	1 (reference)		
High	1.274	0.645, 2.515	0.486

FIGO, International Federation of Gynecology and Obstetrics
